# Status of post-lockdown mental well-being in Bangladeshi adults: A survey amidst COVID-19 pandemic

**DOI:** 10.1371/journal.pgph.0001300

**Published:** 2022-11-28

**Authors:** Mohammad Ali

**Affiliations:** 1 Department of Physiotherapy and Rehabilitation, Uttara Adhunik Medical College and Hospital, Dhaka, Bangladesh; 2 Hasna Hena Pain, Physiotherapy and Public Health Research Center (HPRC), Dhaka, Bangladesh; University of Alberta, CANADA

## Abstract

Lockdown has been recognized as a gold standard measure to limit COVID-19 infection among the general population; however, it has a deleterious impact on their mental well-being. Many studies measured the mental well-being of different population groups during the lockdown period. Nonetheless, very little is known about the mental well-being of the general population when the lockdown has been relaxed or withdrawn in a particular country. Our study aimed to measure the mental well-being of the general population when the lockdown was lifted in Bangladesh. A cross-sectional survey was conducted from December 1, 2020, to February 28, 2021, using both web-based data collection and in-person interview facilities. Data from 3035 general Bangladeshi aged 18 and above were analyzed. Mental well-being was measured using the Warwick-Edinburgh Mental Well-being Scale (WEMWBS) (Registration ID: 518226001). A multivariable linear regression model was employed to find the influential variables after controlling the confounders. The mean well-being score was 43.66. Well-being score was significantly lower among women (slope -2.171, p = <0.001), low-educated (slope -2.485, p = 0.018), and currently not working (slope -2.263, p = <0.001) population. However, we found significantly higher mental well-being scores among those with no comorbidity (slope 3.436, p = <0.001). Though the withdrawal of the lockdown improved the overall mental well-being of the general population, women, low-educated, not working, and the comorbid population were still suffering from low mental well-being problems. Special attention is recommended to address the vulnerable population when discussing the mental health of adult Bangladeshi during and after the COVID-19 pandemic.

## Introduction

Lockdown can be defined as a set of measures aimed at reducing transmission of COVID-19 that are mandatory, applied indiscriminately to a general population, and involve some restrictions on the established pattern of social and economic life [1]. This is one of the most uttered words since the COVID-19 pandemic appeared in the world. World Health Organization (WHO) recognized the lockdown as an effective measure to limit COVID-19 spread. However, lockdown impedes human independence, thus negatively impacting mental health. Previous studies suggested a high prevalence of mental health symptoms among the general population during the lockdown in many countries. For example, a study in the UK found a significantly higher prevalence of mental health symptoms and lower mental well-being scores among the general population at the time of lockdown relative to the pre-pandemic epidemiological data [2]. The prevalence of adverse mental health outcomes in the general Italian population was also significantly high during the COVID-19-related lockdown [3]. A longitudinal study suggested that the French population’s mental health deteriorated during the lockdown [4]. Evidence from Brazil also showed deleterious mental health outcomes due to COVID-19 closer [5]. Data from the USA also suggest poorer mental health among quarantined people [6]. Furthermore, a study in a less COVID-19-affected country, Australia, has also reported a high prevalence of low mental well-being scores among the general population during lockdown [7].

The negative mental impact of lockdown also has been found among the Asian population [8–11]. A subcontinental study suggested a progressively detrimental lockdown effect on various aspects of the psychological health of adult Indians [12]. A similar survey from Pakistan indicated a significant decline in the mental well-being of the general population due to the lockdown [13]. In China, a longitudinal study on the general population’s mental health during the COVID-19 lockdown reported a statistically significant longitudinal reduction of mental well-being scores among observed subjects [14]. In Bangladesh, our previous study found that mental well-being in the general population was worse than the inmates’ mental well-being at the early lockdown stage [15]. In addition to the regional studies, systematic reviews and meta-analyses of cross-sectional and longitudinal studies concluded that COVID-19-related lockdown is strongly associated with the poor mental health of the general population over the world [16–18].

Data from WHO [19] testify that the first case of COVID-19 was detected on March 8, 2020, in Bangladesh. The government has imposed stay-at-home order (lockdown) from March 26, 2020. However, considering the lower infection rate, the Bangladesh government has relaxed the lockdown from the last week of November 2020 to the first week of March 2021. However, until July 2021, there were 1.17 million laboratory-confirmed COVID-19 cases in Bangladesh, while about twenty thousand died [19].

During the lockdown period, numerous cross-sectional studies evaluated the general population’s mental health in several countries, including Bangladesh. However, little is known about the mental well-being status when lockdown has been relaxed or fully withdrawn amidst the COVID-19 pandemic, thanks to the end of the first wave. Our study aimed to measure the mental well-being status of the adult population during the period when the lockdown was lifted in Bangladesh.

## Materials and methods

### Study design and participant

This cross-sectional study data collection commenced from December 1, 2020, to February 28, 2021. Considering the low daily COVID-19 positivity rate, the Bangladesh government lifted the lockdown during this period. However, using a semi-structured questionnaire, we used a cross-platform when collecting 3035 data from Bangladeshi adults aged 18 and above. The same questionnaire was used for both in-person interviews and web-based data collection.

### Sample size calculation

Considering a 95% Confidence Interval (CI), 50% population portion, and 2% marginal error [20, 21], the minimum number of data was calculated at 2401; however, facilities allowed us to collect 3035 data that reduced marginal error to 1.78%.

### The questionnaire

#### Socio-demographic data

The first part of the questionnaire consisted of participants’ socio-demographic information, for example, age, gender, marital status, living location (urban/rural), occupation, educational qualification, and working status. Participants were also asked whether they had comorbidities such as diabetes mellitus, hypertension, asthma, etc.

#### Measurement of mental well-being

The Warwick-Edinburgh Mental Well-being Scale (WEMWBS) was used throughout to provide an appropriate measure of mental well-being. WEMWBS was easy to complete, clear, and unambiguous; it is also popular with practitioners and policymakers [22]. The WEMWBS is a 5-point Likert scale comprising 14 items (e.g., I’ve been feeling optimistic about the future); the answer can be ordered from "none of the time" (1 point) to "all of the time" (5 points). A cumulative score was calculated by totalizing item scores, ranging from 14 to 70. The higher the cumulative score, the higher the level of mental well-being. The scale was not invented through mental illness screening methods, so there is no cut-off point [23]. The questionnaire was used in various studies and validated in several languages and settings, including Bangladesh [15, 22, 24, 25]. Bangla version of the questionnaire was used in this study [26]. Before beginning the survey, a user license for using WEMWBS was obtained (Registration ID: 518226001).

### Recruitment and training of data collectors

Sixteen health science students were recruited to collect and sort data for this study. A 2- day online training program was arranged for the data collectors. However, 12 successful trainees were appointed for further procedures. Six teams of two persons were created. They were taught the techniques for report building and preserving neutrality and were well-informed on ethical issues, privacy concerns, cultural awareness, and risk management for COVID-19 infection. Necessary corrections were made after a pilot study.

### Data collection

#### In-person interview

One thousand five hundred twenty-six data were collected by face-to-face interviews using a paper-based questionnaire. Approximately 1700 public were approached to take part in the interview session. Two districts were selected conveniently from each of all eight divisions of Bangladesh for data collection. However, we employed a two-stage cluster sampling technique to include potential participants for the study. The residential areas, marketplaces, shopping malls, and waiting rooms of large hospitals, diagnostic centers, and bus and rail stations were randomly chosen and processed as a cluster in the first stage. The given data collection sites were collected from the districts’ websites. In the second stage, we chose the participants methodically and conveniently by selecting alternate individuals from diverse groups. Face-to-face data collection technique has been found suitable for similar Bangladeshi studies [20, 27, 28].

#### Web-based data collection

Fifteen hundred and nine data were collected from digital social media platforms using ’Google Forms.’ To disseminate our online questionnaire, we used all commonly used social media platforms in Bangladesh, such as Facebook, WhatsApp, and LinkedIn, and e-mails were sent to professional networks to proclaim the questionnaire. Everyone completing our questionnaire was requested to forward the survey link to their network. Participants were informed about our questionnaire dissemination platforms and were requested to avoid multi-registration. Web-based data collection techniques were also found suitable for collecting data from Bangladesh and other parts of the world during this pandemic for similar Bangladeshi studies [15, 29].

### Ethical consideration

The Ethical Review Committee of Uttara Adhunik Medical College and Hospital has approved this study’s procedures (UAMC-ERC-2020/11). Committee on Publication Ethics guidelines was followed in this study’s designs, data presentations, and citations [30]. Besides, we read and understood the journal’s policies. It is believed that neither the manuscript nor the study violates any of these. Written informed consent was taken from all the participants. Additionally, the voluntary nature of the participation and the purpose of the study was explained before starting the interview.

### Data analysis

Socio-demographic characteristics were described by frequency (percent), mean, and standard deviation. An independent t-test, and one-way ANOVA, were applied to determine the relationship between socio-demographic factors and well-being scores. Afterward, a multivariable linear regression model was demonstrated to determine the predictors of well-being scores. All variables were included in the regression model to adjust the potential confounders, for example, age and living locations. Factors at the 5% significance level were defined as statistically significant. The Cronbach’s alpha value for the items of WEMWBS in this study was 0.891, which indicates excellent internal consistency. Data have been analyzed using The Statistical Package for the Social Science (SPSS) software version 23.0, SPSS Inc., Chicago, IL, USA.

## Results

### Socio-demographic characteristics in comparison to the well-being scores

This study was dominated by the men (60%) and younger (20–29 years) age group (61%) population. About 67% of the subjects lived in an urban area, while 62% were unmarried. Regarding occupation, half of the total participants were from working groups. About 44% of the participants graduated, and just below half of the participants were currently working from home or at the office. When asked about comorbidities, only 18% of participants answered that they have at least one chronic disease, such as hypertension.

The mean well-being score for all subjects was 43.66, with a standard deviation (SD) of 12.56. However, this study found that the well-being score was significantly lower among women (p = <0.001). Similarly, homemakers and businesspersons reported significantly lower well-being scores (p = <0.001). Lower educational qualification (p = 0.010) and currently not working (p = <0.001) status were significantly associated with lower well-being scores. Also, lower well-being scores have been found in subjects suffering from comorbidities (p = <0.001) than in healthy ones. Details are presented in Table 1.

**Table 1:** Participants’ characteristics and association with the mental well-being score.

Factors	N (%)	Mean	Standard deviation	p-value
Total	3035 (100)	43.66	12.56	.
*Sex*				**<0.001**
Female	1199 (39.5)	42.42	12.53	
Male	1836 (60.5)	44.46	12.52	
*Age*				0.512
≤19	226 (7.4)	44.04	14.11	
20–29	1866 (61.5)	43.79	12.94	
30–39	651 (21.4)	43.69	11.88	
40–49	211 (6.9)	42.51	09.36	
≥50	81 (2.7)	42.20	11.67	
*Living location*				0.371
Rural	984 (32.4)	43.95	12.28	
Urban	2051 (67.6)	43.51	12.69	
*Marital Status*				0.320
Unmarried	1906 (62.8)	43.83	12.87	
Married	1065 (35.1)	43.26	11.99	
Others	64 (2.1)	45.11	12.67	
*Occupation*				**<0.001**
Student	1174 (38.68)	43.11	13.27	
Homemaker	176 (5.8)	41.18	12.20	
Business	250 (8.2)	42.04	11.79	
Healthcare	491 (16.2)	44.38	11.31	
Non-Govt. service	548 (18.1)	45.08	12.26	
Govt. service	188 (6.2)	45.71	12.33	
Unemployed	208 (6.9)	43.42	12.83	
*Educational Qualification*				**0.010**
Schooling 6–10	241 (7.9)	41.59	13.72	
Higher Secondary	641 (21.1)	43.16	13.11	
Graduate	1356 (44.7)	44.32	12.18	
Post-graduate	797 (26.3)	43.57	12.31	
*Current working status*				**<0.001**
No	1647 (54.3)	42.55	12.74	
Yes	734 (24.2)	45.17	11.91	
Work from Home	654 (21.5)	44.75	12.57	
*Comorbidity*				**<0.001**
No	2487 (81.9)	44.28	12.79	
Yes	548 (18.1)	40.84	11.12	

### Results from multivariable linear regression

Table 2 depicts results from the multivariable linear regression model. The results present the slope estimate, the slope’s 95% confidence interval (CI), and the corresponding p-value. The model revealed that gender, educational qualification, current working status, and comorbidity significantly affect mental well-being scores. Compared with the men, women had significantly lower well-being scores with a slope of -2.17 (95% CI -3.17 to -1.18; p = <0.001). Participants with lower education (schooling 6–10) showed significantly lower mental well-being than the post-graduate group, with a slope of -2.5 (95% CI -4.54 to -0.043; p = 0.018). On the other hand, participants who had no comorbidity were in a significantly better mental well-being state (slope 3.5, 95% CI 2.20 to 4.68; p = <0.001) than the comorbid group. Results from the assumption of fitting linear regression tests can be found in S1 File.

**Table 2:** Results from a multivariable linear regression model with well-being score as the outcome. (The positive slope means better mental well-being).

Factors	Slope	95% Confidence Interval		p-value
		Lower bound	Upper bound	
*Sex*				
Female	-2.171	-3.166	-1.176	**<0.001**
Male	Reference	.	.	.
Others				
*Age*				
≤19	2.167	-1.544	5.877	0.252
20–29	-0.125	-3.301	3.050	0.938
30–39	-0.653	-3.748	2.442	0.679
40–49	-1.078	-4.445	2.288	0.530
≥50	Reference	.	.	.
*Living location*				
Rural	0.637	-0.332	1.606	0.197
Urban	Reference	.	.	.
*Marital Status*				
Unmarried	0.613	-3.222	4.449	0.754
Married	0.324	-3.470	4.117	0.867
Others	Reference	.	.	.
*Occupation*				
Student	-1.170	-3.107	0.767	0.236
Homemaker	0.157	-2.614	2.928	0.911
Business	-1.443	-3.926	1.041	0.255
Healthcare	0.518	-1.644	2.679	0.639
Non-Govt. service	1.038	-1.087	3.163	0.338
Govt. service	1.775	-0.836	4.386	0.183
Unemployed	Reference	.	.	.
*Educational Qualification*				
Schooling 6–10	-2.485	-4.540	-0.430	**0.018**
Higher Secondary	-0.017	-1.473	1.439	0.982
Graduate	1.031	-0.147	2.208	0.086
Post-graduate	Reference	.	.	.
*Current working status*				
No	-2.263	-3.411	-1.114	**<0.001**
Yes	-0.005	-1.399	1.389	0.994
Work from Home	Reference	.	.	.
*Comorbidity*				
No	3.436	2.196	4.676	**<0.001**
Yes	Reference	.	.	.

## Discussion

To the best of our knowledge, this is the first study that measured mental well-being among adults when the COVID-19-related lockdown was lifted in Bangladesh. This study revealed moderate well-being scores among the surveyed population. Well-being scores were significantly lower among women, homemakers, unemployed, low-educated, and comorbid participants. However, gender, educational qualification, current working status, and comorbidity independently predicted well-being scores.

Compared to our previous study conducted in a similar population at the early stage of lockdown using the same questionnaire, this study reported significant improvement in the overall WEMWBS scores (38.4 vs. 43.66) [31]. This post-lockdown study results align with the comparative analysis that measured the general population’s pre and post-pandemic mental health [2]. However, we found disappointing scenarios when we compared the mental well-being scores in this study with the different population groups of other countries. For example, a survey conducted among Irish prisoners found well-being scores similar to those we saw in the current study [32]. On the other hand, previous studies suggested that the general population in Spain, Denmark, and England showed much better mental well-being during the standard time [24]. These comparisons added evidence of the long-lasting pandemic’s detrimental impact on the general population’s mental health, even in the post-lockdown situation.

Even though the fatality rate is twice for men as for women, the COVID-19 pandemic has affected women’s mental health more than men’s [33]. Our study also revealed that the mental well-being score in Bangladeshi women was significantly lower than that of their counterparts. Generally, women’s risk of depression, anxiety, and post-traumatic stress disorder is considerably higher [34]. In addition, it is reported that domestic and workplace violence incidences have increased in many countries after the viral outbreak [35]. However, in the context of the pandemic, the worsening of gender-based violence may not receive the attention warranted. Similarly, a lack of adequate domestic and emotional support can have consequences on women’s mental health. All these risk factors could explain women’s higher rate of mental health problems during the COVID-19 pandemic.

Pandemic-induced economic turmoil affects the underprivileged population worldwide [11, 36]. Our study found significantly lower mental well-being scores among low-educated participants who were not working at the time of the survey. We found similar results when evaluating Bangladeshi people’s well-being at the early lockdown stage [31]. However, the results of both studies suggested that the mental well-being scores of the not working population were similar during and after the lockdown. A previous study conducted by other authors also suggested poorer mental health among unconsidered Bangladeshis [37]. Additional study is required to understand the long-term effect of lockdowns among the underprivileged population in low and middle-income countries (LMICs) such as Bangladesh.

Evidence suggests that the COVID-19 death rate is considerably higher among comorbid patients. Underlying medical conditions, especially hypertension and diabetes, are responsible for a worse prognosis in COVID-19 [38]. Similarly, people suffering from chronic obstructive pulmonary disease or other respiratory illnesses are also at higher risk for severe illness from COVID-19 [39]. The risk of contracting COVID-19 in patients with comorbidities is also much higher than in their counterparts [38]. Given the higher risk of contractibility and worse prognosis, comorbid people are under much mental pressure during the pandemic time. Our study found a highly significant lower mental well-being score among the comorbid population during the survey time.

Universal limitations of the study’s cross-sectional nature and limitations of the WEMWBS scale must be admitted for this study. Furthermore, data regarding family income, food security, dietary habits, and physical activity were not taken for this study, limiting the result. This study was dominated by urban, male, and younger participants; thus, caution should be taken when generalizing the findings. Despite the limitations, this study provided baseline data on the mental well-being of LMICs’ general population when lockdown has been paused. These data also provide opportunities to compare the general population’s mental health at the time of lockdown, and the lockdown has been halted.

## Conclusion

This study suggested that the withdrawal of lockdown improves the mental well-being status of the general Bangladeshi population. However, the mental well-being of women, homemakers, low-educated, unemployed, and people with comorbidities remained in the poorer state despite the lockdown restrictions relaxed. Special attention must be considered for these population subgroups when approaching mental health aids for the general population of LMICs, such as Bangladesh, during and after the pandemic.

## Supporting information

S1 File (DOCX)
