# Supplementary material for: Status of post-lockdown mental well-being in Bangladeshi adults: A survey amidst COVID-19 pandemic
Source: PLOS Glob Public Health. 2022 Nov 28;2(11):e0001300. doi: 10.1371/journal.pgph.0001300 (PMC10021766; doi:10.1371/journal.pgph.0001300)
Supplement: S1 File — (DOCX) [file pgph.0001300.s001.docx]

**Assumption of fitting linear regression:**

The requirments of linear regression:

1. Normally distributed error components: Quantile-quantile (Q-Q) plot depicts that the data was distributed normally (**Fig A**).
2. Absent of heteroskadisticity: **Fig B** shows that there are no heteroskadisticity, the variance of the residuals are constant across the predicted values.
3. Multicollinearity statistics:

Multicollinearity statistics suggested that tollerance (T= 1-R^2^) are not <0.1. On the other hand, variance inflation factor (VIF) (VIF= 1/1-R^2^) are not >10. (**Table A**).


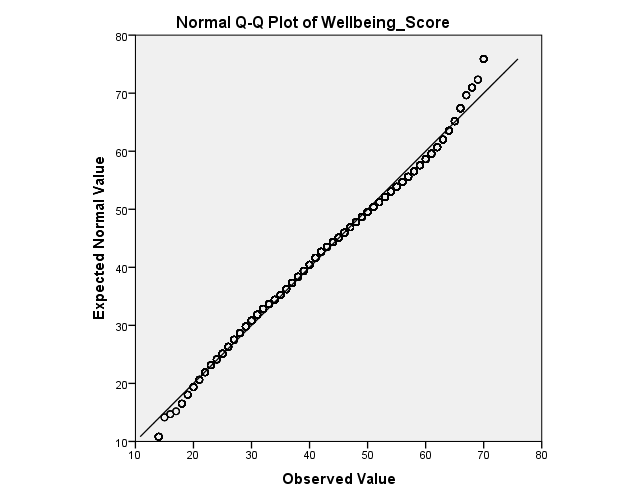


Fig A: Quantile-quantile (Q-Q) plot


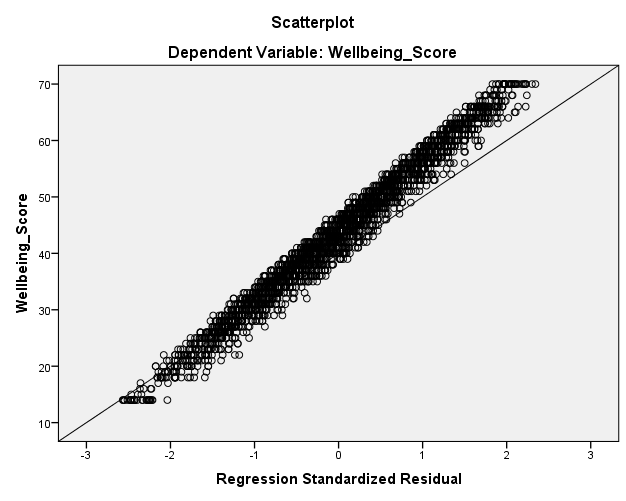


Fig B: Homoskedasticity

Table A: Multicollinearity statistics

| Model | | Collinearity Statistics | |
| --- | --- | --- | --- |
|  |  | Tolerance | VIF |
|  |  |  |  |
|  | Living location | .976 | 1.024 |
|  | Sex | .947 | 1.056 |
|  | Marital status | .691 | 1.446 |
|  | Age | .596 | 1.678 |
|  | Occupation | .774 | 1.291 |
|  | Education | .846 | 1.182 |
|  | Working status | .992 | 1.008 |
|  | Comorbidity | .888 | 1.126 |
